# Experiences of Older Adult Parents of Adult Persons with Autism Spectrum Disorder

**DOI:** 10.1093/geroni/igaf122.1084

**Published:** 2025-12-31

**Authors:** Yarin Cohen, Robert Hock, Abigail Reza, Emmanuella Asabea Twum

**Affiliations:** University of South Carolina, Columbia, South Carolina, United States; University of South Carolina, Columbia, South Carolina, United States; University of South Carolina, Columbia, South Carolina, United States; University of South Carolina, Columbia, South Carolina, United States

## Abstract

For parents of adult children with ASD, aging is often accompanied by caregiving that creates time, financial, and lifestyle constraints. Previous research has found that support networks decrease after individuals become adults and leave school, while parents, facing health and aging challenges, may hesitate relying on family for support, risking future care crises. Yet little is known about how these parents navigate the intersection of aging and persistent caregiving. This qualitative study aimed to addressing the unique challenges and described needs, while identifying coping mechanisms that promote older adult caregiver well-being. Semi-structured interviews were conducted with 13 participants (age 54-72, 3 males, 10 females) caring for adult children with ASD (age 25-36) exploring caregiving in later life, service experiences, family relationships, and preparing for the future. These interviews were recorded and transcribed for analysis using a thematic approach. Analysis revealed five key themes: Feeling disconnected; experiencing service gaps after school transitions; anxiety about future care arrangements; prioritizing child’s needs above their own; ongoing advocacy challenges. Parents often struggle finding mental health support, may feel isolated, and grapple with planning for their child’s future care, a process that can be both emotionally distressing and logistically complex. However, they also find strength in established routines and skills, highlighting both challenges and resilience in long-term caregiving. Enhanced support systems are needed to assist adults with ASD in transitioning to adulthood, including accessible guidance on future planning and establishing peer networks where older caregivers can share valuable knowledge with younger families.

